# Management of acute and post-operative pain in chronic kidney disease

**DOI:** 10.12688/f1000research.2-28.v3

**Published:** 2013-04-05

**Authors:** Malvinder S Parmar, Kamalpreet S Parmar

**Affiliations:** 1 Northern Ontario School of Medicine, Laurentian & Lakeland Universities, Ontario, P3E 2C6, Canada; 2 University of Queensland, Brisbane, Queensland, QLD 4072, Australia

## Abstract

Chronic kidney disease is common and patients with many co-morbid conditions frequently have to undergo surgical procedures and, therefore, require effective pain management. The pharmacokinetics of various analgesic agents are not well studied in patients with chronic kidney disease and the risk of accumulation of the main drug or their metabolites, resulting in serious adverse events, is a common scenario on medical and surgical wards. It is common for these patients to be cared for by 'non-nephrologists' who often prescribe the standard dose of the commonly used analgesics, without taking into consideration the patient's kidney function. It is important to recognize the problems and complications associated with the use of standard doses of analgesics, and highlight the importance of adjusting analgesic dosage based on kidney function to avoid complications while still providing adequate pain relief.

***Illustrative clinical scenario:*** *- an example to illustrate the problem.*

*A 63-year-old man with diabetes and end stage renal disease on maintenance continuous ambulatory peritoneal dialysis was admitted to the hospital with gangrene of the left foot and underwent left below-knee amputation under spinal anesthesia. Postoperatively, he was given meperidine (Demerol) 50–75 mg intramuscularly every 3 hr, as required, by the orthopedic surgeon, but this was found to be ineffective in controlling pain. The anesthetist ordered morphine 10–15 mg intramuscularly or subcutaneously every 3 hr as required (pro re nata) in the evening. He received 325 mg of meperidine in the first 24 hr and additional 200 mg of meperidine next day (total 525 mg) and 55 mg of morphine within the first 48 hr after surgery. 48 hr after surgery, he was found to be confused and somnolent. On examination the patient was drowsy and somnolent, but arousable with twitching of arms, and complained of mild pain at the stump site when awakened. He had labored breathing with a respiratory rate of 8 breaths per minute. An arterial blood gas showed pH of 7.19, pCO _2_ of 60, pO _2_ of 86 and oxygen saturation of 95%. His mental state improved transiently with intravenous naloxone, confirming opioid effect. How could this situation have been prevented while effectively providing pain relief?*

Chronic kidney disease (CKD) is common ^1^ and is regularly accompanied with various co-morbidities. Often these patients with various co-morbidities undergo operative procedures and require analgesics. The most-responsible physician providing the care to these individuals during their hospitalization is often a non-nephrologist. Opioids are commonly used in the management of post-operative pain, and often a standard dose is used by the health-care providers without taking into consideration kidney function ^2^. In North America, morphine and meperidine (Demerol) still remain the commonly used parenteral agents, and acetaminophen with codeine (Tylenol #3) a commonly used oral agent for management of post-operative pain in patients with ^3^ and without renal failure. Prolonged central nervous system and ventilatory depression due to morphine and other opioids have been known for over a century in patients with kidney failure ^4– 10^ and the various contributing factors are outlined in Table 1. Despite the increased potential for respiratory depression, especially in patients with CKD, life-threatening cases of opiate toxicity continue to occur not only in patients with kidney disease ^11^, but also in patients without kidney disease ^12, 13^. It is important to highlight the issue of central nervous system (CNS) and respiratory depression with opioid analgesics in patients with CKD and formulate strategies to prevent these complications, while providing effective pain relief.

**Table 1.** Factors contributing to opioid toxicity in CKD.

Factors contributing to opioid toxicity in chronic kidney disease		
Patient factors		Exogenous factors
Independent of kidney function • Age – extremes of age, increases sensitivity • Gender – women more sensitive • Genetic – absence of A118G SNP* • Route of administration§ • Enterohepatic circulation • Hypoalbuminemia • Concomitant illness(es) – Sepsis, liver disease	Dependent on kidney function • Decreased clearance – accumulation of parent drug and active metabolites • Decreased threshold • Change in volume of distribution (V _d_) • Change in protein binding • Impaired ventilatory response tocarbon dioxide in CKD	Drug-drug interactions Cytochrome P450 2D6 system ^@^ P-glycoprotein inhibitors¶ Concomitant antibiotic useº

* A118G polymorphism protects against M6G-related opioid toxicity ^28^.

§ Routes that use first pass metabolism results in higher production of metabolites than those that bypass it.

¶ P-glycoprotein inhibitors increase uptake of M6G across blood brain barrier ^16, 17^.

º Concomitant antibiotics by altering bacterial flora reduces bacterial glucuronidase, resulting in reabsorption of M6G.

^@^ Extensive 2D6 metabolism may occur in up to 1/3 ^rd^ of patients of east African heritage – increasing the risk of opioid overdose from codeine.

In addition to the post-operative pain, patients with CKD may have underlying chronic pain that is often multi-factorial ^14^ i.e., ischemic pain from peripheral vascular disease, neuropathic pain of polyneuropathy (diabetes), bone pain from osteoporosis or dialysis-associated amyloidosis, and musculoskeletal pain that may or may not be related to kidney disease. Post-operative pain is often nociceptive, whereas chronic pain may be nociceptive, neuropathic or mixed. It is also important to recognize that depression is common in CKD ^15^ and that it may complicate the management and patient’s ability to cope with pain ^16^.

## Assessment of pain

Assessment of pain requires detailed history to elucidate the cause of pain, its location, nature (acute, chronic or acute and chronic), intensity and its impact on physical, social and emotional functioning ^17^.

## Spectrum of pain

Acute pain is a protective and an adaptive response to injury, whereas chronic pain or the persistence of pain beyond the healing phase is a maladaptive ^18^. Acute pain results from direct stimulation of sensory neurons, called nociceptors. Nociceptors have two types of axons, the rapidly conducting thinly myelinated Aδ fibers that mediate the initial phase of acute pain that is extremely sharp pain, and the more slowly conducting unmyelinated C fibers mediate the second phase of acute pain that is more prolonged and less intense following injury. Nociceptors can be stimulated by mechanical, thermal, chemical and inflammatory stimuli ^18^.

Pain lasting longer than 3 months or beyond the duration required for complete tissue healing is called chronic pain. It may be nociceptive, resulting from prolonged tissue injury with persistent activation of nociceptors, neuropathic resulting from a lesion or a process affecting the somatosensory system or mixed processes.

## Intensity of pain

Pain intensity may be measured by one of the major pain rating scales including verbal, numerical and visual analogue scales. However, a numerical rating scale comprising of a range of numbers from 0 to 10, is the most widely used system in clinical practice and is generally based on a subjective 10-point scoring system, where 0 denotes the absence of pain and 10 the worst pain imaginable ^19^.

## Pain management

Effective pain management requires a multifaceted approach with an understanding of the type (nociceptive, neuropathic or mixed), underlying cause, assessment of intensity and duration of pain ^17^. In addition, assessment of concomitant medications and co-morbidities is important when selecting analgesic agent(s). Adjuvant analgesics and non-pharmacological methods of pain control are important to consider in such circumstances ^17^. We review the strategies to manage acute pain in CKD with main focus on the use of opioids since most of the challenges occur in the use of opioids in CKD.

**Figure 1.**
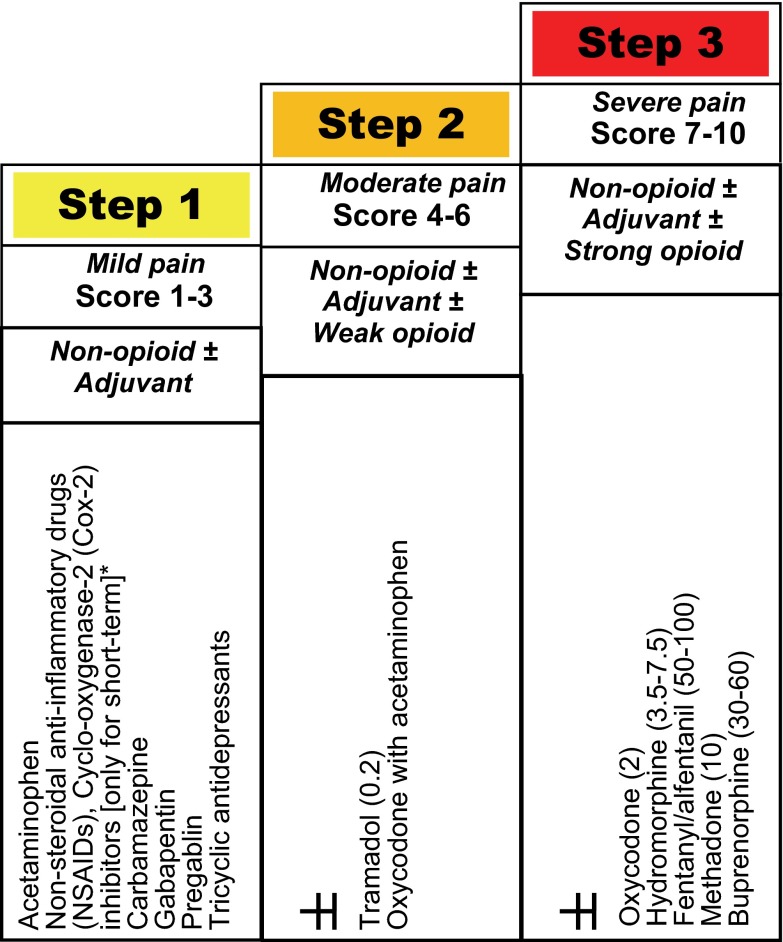
Modified WHO analgesic ladder in patients with chronic kidney disease [adapted from references ^17, 18^], with relative potency compared to morphine (1) of commonly used opioids in parenthesis. *Non-steroidal anti-inflammatory agents, including cyclo-oxygenase-2 inhibitors should be avoided, except for acute pain with close clinical observation.

In 2005, a modified World Health Organization (WHO) step-wise approach was proposed for pain management in patients with CKD ^20^ ( Figure 1) and this approach was found to be effective in acute pain control in >90% of hemodialysis patients, at least short-term, in a 4-week study ^21^. This approach is important to follow to achieve ‘balanced analgesia’ by effectively utilizing ‘co-analgesics’ ( Box 1).

Box 1. **Glossary.**

Opiate: Naturally occurring alkaloid i.e., morphine or codeine.

Opioid: Any natural or synthetic compound that has morphine-like properties.

Balanced analgesia: Principle of combining different classes of analgesics to optimize efficacy while minimizing side-effects.

Co-analgesic: A drug administered alongside opioids enabling a reduction in opioid dose.

Adjuvant: A substance that helps and enhances the pharmacologic effects of an agent (analgesic).

## Non-opioid analgesics

**Acetaminophen:** Has potent analgesic and antipyretic properties and is effective in various acute and chronic painful syndromes. It is the analgesic of choice in the elderly and in patients with kidney disease ^22^ and should be considered in all patients (except in patients with hepatic insufficiency) experiencing pain, regardless of pain level, until pain is relieved or up to a maximum dose of 3,000 mg a day ^23^ or 2.6 g/d in high-risk patients ^24^ (malnourished or alcoholic). It often does not require dose adjustment in CKD but some authors recommend increasing dosing interval from every six to eight hours when GFR is <10 mL per minute ^25^. Acetaminophen is underutilized in the peri-operative period and when prescribed it is often in an "on demand" basis rather than "by the clock" ^17^. We suggest consider prescribing at least half of total daily dose “by the clock” (opinion MSP) e.g., Acetaminophen 500 mg four times a day and remaining dose could be given “on demand” or in combination with other agents.

**Anti-inflammatory agents:** Nonsteroidal anti-inflammatory drugs (NSAIDs) or cyclo-oxygenase (COX-2) inhibitors block prostaglandin synthesis and, when used as co-analgesic, reduce opioid need by 30–50%. However, because of their gastro-intestinal and cardio-renal side effects, they should be avoided for prolonged use in CKD, as these agents may decrease renal blood flow and may precipitate acute renal failure and can cause life-threatening hyperkalemia. However, if used, it should be for short term management (3 to 7 days) of acute pain and consider short-acting agents ^26^ with careful monitoring of renal function and serum potassium.

## Adjuvant analgesics

Adjuvant analgesics are medications with a primary indication other than pain that may possess analgesic activity or modify pain response. These are often helpful for management of chronic neuropathic pain. Examples include cabamezapine, gabapentin, and pregabalin. These agents are not required for management of acute pain. However, CKD patients may have associated underlying chronic neuropathic pain that could affect acute pain response or management. Therefore, some patients may already be taking these agents for control of their 'chronic pain’ and these agents may be stopped inadvertently in the peri-operative period. If this is the case, adjuvant analgesics should be resumed when possible, with considerations being taken for the possible drug-drug interactions when prescribing opioid analgesics for acute pain. It is important to understand that the dose of these agents (adjuvants) may also need to be reduced because of renal dysfunction.

## Opioids

The relative potency of commonly used opioids is shown in Table 2. As most opioids or their metabolites are excreted by the kidneys, dosage adjustment is often required when estimated glomerular filtration rate (eGFR) falls below 50 ml/min or during stages 3b, 4 and 5 of CKD, and for patients on dialysis (Table 2) ^27, 28^. In addition to CNS and respiratory depression, constipation is a common side effect that is important to identify and manage, especially in patients on peritoneal dialysis, as constipation is the common cause of catheter malfunction ^29^, and appropriate laxatives should be used in conjunction with treatment.

**Table 2.** Recommendations for commonly used opioids in CKD. The dose adjustment based on eGFR shown is the percentage of recommended normal dose, 75% of recommended dose means 25% dose reduction, 50% of recommended dose means 50% dose reduction and 25% of recommended dose means 75% dose reduction.

IN CKD	Opioid	Comments	Dialyzability	Dose adjustment based on eGFR				Recommendation(s)
				Normal	CKD (eGFR/ml)			
					>50	10–50	<10/RRT	
PARENT COMPOUND	Morphine	Metabolites accumulate in renal failure, CNS and Respiratory depression, 20–25% of patients may tolerate well	Yes, but M6G slowly re-equilibrates across the blood brain barrier, delaying the response to hemodialysis; Very small amount removed by CVVH or CVVHD		75–100% 2.5–5 mg q6h	50–75% 2.5–5 mg q6-8h	25–50% small dose 1.25–2.5 mg q8-12h	Short-term use Avoid standing order Reassess dose q24-48h Not recommended for long-term use
RECOMMENDED/USE WITH CAUTION	Hydromorphone	Metabolite H3G accumulates in renal failure, has no analgesic activity but possibly neuro-excitatory	Yes	1.3 mg q6h	100%	75% Start with 0.5 mg q6-8h	50% Start with 0.5 mg q6-8h	**RECOMMENDED, use carefully**
	Fentanyl	Inactive metabolite, highly protein bound, large volume of distribution (V _d_). CNS and Respiratory depression reported with infusion and Transdermal patch	No	25–100 mcg q4-6h SC	100%	75%	25–50%	**RECOMMENDED**, starting dose 25–50 mcg q4-6h SC. Transdermal patch takes 3 days to reach steady state Do NOT use patch in opioid naïve patients
	Tramadol	20% protein bound	Slowly removed , 50% clearance by HD	50–100 mg q6h	100%	50% q12h	50% q12h	Use with caution, dose after hemodialysis on dialysis days
	Oxycodone	50% protein bound	To some extent	5–10 mg q6h	100% q6h	50–75% q8h	25–50% 18–12h	Limited evidence, use with caution
	Buprenorphine	Partial opioid agonist, ceiling analgesic effect, 96% protein bound	unlikely	0.3 mg IM/IV 8–16 mg S/L		Reduce dose and increase interval	Reduce dose and increase interval	Use with caution
NOT RECOMMENDED	Codeine	Both codeine and its metabolites accumulate in renal failure			100% q6h	75% q8h	50% q12h	**Not recommended in dialysis patients**
	Dihydrocodeine		NA	NA	NA	NA	NA	**Not recommended**
	Meperidine	Toxic metabolite, normeperidine accumulates in renal failure and can cause seizures	Yes		75–100%	50%	Avoid	**AVOID**
	(Dextro)Propoxyphene	Toxic metabolite, norpropoxyphene accumulates in renal failure, can cause seizures, hypoglycemia and cardiac conduction disturbances	No		Avoid	Avoid	Avoid	**AVOID**

**Morphine:** Morphine is the most studied opiate in kidney disease. It is primarily an agonist at the µ-receptors with pain relief at µ _1_-receptors, and causes respiratory depression and constipation through its agonist action at µ _2_-receptors ^14^. Morphine is metabolized in the liver to several metabolites ^30^. Of these diamorphine and morphine-3-glucuronide (M3G) do not bind to opiate receptors, whereas normorphine and morphine-6-glucuronide (M6G) do, with M6G being approximately 10-fold more potent than morphine ^31^. All metabolites are excreted in the urine along with about 10% of the parent compound. M6G accumulates in kidney failure and causes CNS and respiratory depression ^32^. M6G crosses the blood brain barrier (BBB) slowly, and once it crosses the BBB, the CNS effects may be prolonged and may persist after stopping morphine or after hemodialysis, as the M6G slowly re-equilibrates across the BBB back into systemic circulation ^6^. Transport of M6G across the BBB is an active process mediated by 170-kd P-glycoprotein. P-glycoprotein is an ATP-dependent carrier system that acts as an efflux pump to transport chemicals or metabolites from endothelial cells to vascular compartment and inhibition of this results in an increase of M6G in the brain ^33, 34^. The analgesic and respiratory depressant effects are related to serum concentrations of both morphine and M6G ^31^. A dosage reduction of 25%, 50% and 75% is recommended in patients with mild (stage 3), moderate (stage 4) and severe kidney failure (stage 5) respectively. However, 20–25% of patients may tolerate the full dose of morphine without ill-effects because of *A118G* polymorphism ^35, 36^.

**Meperidine:** Normeperidine, an active metabolite of meperidine, is neurotoxic and accumulates in renal failure and may cause a range of neuroexcitatory effects ranging from irritability to agitation to seizures ^37^. Although normeperidine is removed by hemodialysis ^38^, its regular administration is ***contraindicated*** in patients with any degree of renal failure ^39^.

**(Dextro)Propoxyphene:** It is no longer available in North America and its metabolite norpropoxyphene is excreted by the kidneys and accumulates in renal failure ^39^. This results in CNS and respiratory depression, cardiac conduction disturbances and hypoglycemia. Both the parent drug and its metabolite are not removed by hemodialysis and is ***contraindicated*** in patients with renal failure ^39^.

**Hydromorphone:** Hydromorphone is an analogue of morphine with shorter duration of action and with excellent efficacy in moderate to severe pain. It is five to seven times potent than morphine. It is eliminated by both hepatic (60%) and renal routes ^40^. Hydromorphone doesn’t accumulate in renal failure because of its rapid conversion to its less-potent metabolite hydromorphone-3-glucuronide (H3G) that accumulates in renal failure but is effectively removed by hemodialysis ^41^. However, typical opioid adverse effects have been reported in patients with renal failure ^42^, but experience suggests efficacy without excess toxicity, if monitored carefully ^43^.

**Codeine/Dihydrocodeine:** Codeine is metabolized by the liver to a variety of active metabolites (codeine-6-glucuronide, norcodeine, morphine, M3G, M6G, and normorphine) that are renally excreted ^44^. The half-life of codeine is prolonged 5-fold in hemodialysis patients ^45^. Codeine and its metabolites accumulate in renal failure and can cause hypotension and CNS and respiratory depression ^46, 47^. A similar elimination pathway is proposed for elimination of dihydrocodeine, although this has not been fully evaluated ^39^. Dihydrocodeine can cause prolonged narcosis after therapeutic doses in patients with acute ^48^ or chronic renal failure ^49^. Therefore, these agents should be used with caution in patients with renal failure. A reduction in dose by 50% is suggested for codeine in renal failure ^39, 45^ and chronic use should be avoided.

**Oxycodone:** Oxycodone is a strong opioid with a higher oral bioavailability. It is metabolized by the liver to active metabolites – noroxycodone and oxymorphone ^50^. Approximately 19% of the drug is excreted unchanged in the urine. The clearance of oxycodone and its metabolites is reduced in renal failure ^50^ and results in an increase in plasma concentration by 50% and prolongation of its half-life by an hour ^50^. There is lack of consensus of its use in CKD because of varied case reports of toxicity and good tolerance. When required, use cautiously and start with a lower dose ^51^.

**Buprenorphine:** Buprenorphine is a long-acting semi-synthetic partial agonist with the advantage of having less respiratory depression and hypotension ^52, 53^ but has a ceiling effect ^54, 55^. There is no significant difference in the clearance of the drug in patients with normal or impaired renal function. It has limited oral bioavailability because of an extensive first-pass metabolism. Sublingual, transdermal or parenteral administration is required. It is extensively metabolized by the liver with less than 30% renal excretion. The metabolite norbuprenorphine may accumulate in renal failure that has minor analgesic activity. Buprenorphine is 96% protein bound so is not dialyzable ^56^.

**Fentanyl, Alfentanil, and Sufentanil:** Fentanyl, Alfentanil and Sufentanil are potent opioid receptor agonists, with a rapid onset of action and shorter duration of action. Fentanyl is rapidly metabolized in liver to inactive metabolites. Less than 10% of the parent drug is excreted in urine with no significant accumulation in CKD, but there is considerable inter-patient variability in fentanyl pharmacokinetics when given as a continuous infusion or via transdermal route. However, no dosage modification is necessary in patients with renal failure when fentanyl is given as a bolus. However, prolonged sedation is observed in critically ill patients when fentanyl is given as a continuous infusion, as its half-life increases to 25 hr ^39^ due to saturation of its distribution sites, independent of renal function. Accumulation of fentanyl may occur when given by infusion ^39^ or transdermal patch. Its clearance may be altered in advanced kidney disease ^57^ and prolonged sedation and ventilatory depression have been observed in patients with end stage renal disease following fentanyl infusion ^39^. Fentanyl is highly protein bound (80–86%), has a high volume of distribution, and removal by hemodialysis is negligible ^58, 59^. Opioid-naïve patients should not be initiated on transdermal fentanyl because of its variable absorption.

**Tramadol:** Tramadol is an opioid analgesic that also inhibits serotonin and noradrenaline reuptake. It is extensively metabolized by the liver and 30% of the parent drug and 60% of active metabolites are excreted in the urine ^60^. It is effective for both nociceptive and neuropathic pain and has the advantage of less sedation and respiratory depression compared to other opiates. A common side effect is nausea. A rare but important side effect is seizures, especially in patients taking medications that lower the seizure threshold, like selective serotonin reuptake inhibitors (SSRI). In addition, tramadol may precipitate serotonin syndrome ^61^ (a potentially life-threatening drug-interaction manifested by excess serotonin effect resulting in cognitive, autonomic and somatic effects) in patients taking SSRI ^62^. In advanced CKD, the elimination half-life of tramadol may double ^60^ and the dose should be decreased to 100 mg every 12 hr in patients with eGFR of 30 ml/min and 50 mg every 12 hr when eGFR is <10 ml/min. Tramadol is significantly removed by dialysis and should be administered after hemodialysis on dialysis days ^63^.

**Methadone:** Methadone is a synthetic opioid with five to 10 times the potency of morphine, is highly protein bound (70–87%) and has a half-life of 30 hr. Approximately 20% after a single dose is excreted unchanged and about 30% as inactive metabolites in the urine ^39^. There is a 3-fold increase in the excretion of metabolites on chronic dosing, suggesting enhanced metabolism ^64^. The removal by hemodialysis is insignificant ^65^. Although in 2 patients with CKD the plasma concentrations were no higher than patients with normal renal function ^66^, there is a well-described risk of accumulation and toxicity with methadone, even in patients with normal renal function, so experienced specialist supervision is warranted. A dose reduction of 50% to 75% is suggested in patients with renal failure, but it is difficult to use in patients with CKD because of its long half-life and wide inter-individual variation in clearance ^65^. In some countries, methadone may require a specialist in pain management with a special license to prescribe it, and that may limit its use in acute pain management. However, methadone should not be used in opioid naïve patients.

**Table 3.** Pain management in CKD.

Pain intensity	WHO analgesic step	Agent(s) of choice	Comments
*Mild pain* Score 1–3	*Non-opioid* ± *Adjuvant*	Acetaminophen 500 mg q6h (by the clock) and 325 mg every 6–8h as required	Not to exceed total dose of 3g in 24-hours; If unable to take orally, then consider suppository NSAIDs/Cox-2 inhibitors are *not recommended*, but may consider, for short-term ONLY, under close observation
*Moderate pain* Score 4–6	*Non-opioid* ± *Adjuvant* ± *Weak opioid*	± Tramadol 50 mg every 12h, if required or Oxycodone with acetaminophen every 8–12h	Maximum dose 200 mg in stage 4 CKD and 100 mg in stage 5 CKD, dose after dialysis
*Severe pain* Score 7–10	*Non-opioid* ± *Adjuvant* ± *Strong opioid*	± Hydromorphone 1.3 mg every 8h or Oxycodone 2.5 mg every 8–12h or Fentanyl 25–50 mcg SQ every 4–6h or Buprenorphine 0.3 mg every 6h IM/IV 8–16 mg sublingual daily Transdermal Patch – 5 mcg/hr–20 mcg/hr or Morphine 1.25–2.5 mg every 8–12h (for short-term use)	Use laxatives to avoid constipation, when using opioids Do not write standing (or long-term) orders for opioids. Reassess the need and dose of opioids every 24–48 hours Monitoring for CNS and respiratory effects is required for protracted periods. Fentanyl and methadone are highly protein bound and not dialyzable. Avoid fentanyl transdermal patch and methadone in opioid-naïve patients

## Summary

Patients with CKD have a high burden of co-morbidities that modulate pain response, and when these individuals undergo operative procedures, standard orders for pain control can be written without taking into consideration the patient's kidney function. Unfortunately, the data on the effects and tolerability of various analgesic agents are limited in such individuals and, given the lack of data, the recommendations are mainly based on expert opinion ^17, 67^ in patients with CKD with ^68^ and without dialysis ^43^. The general principles of pain assessment and management should be adopted and analgesia should be prescribed incorporating the modified WHO analgesic ladder ^20^ in patients with CKD, with close and frequent monitoring for side effects of drug or its metabolite accumulation. The nephrologist and a pharmacist should be involved in the management of these patients. The non-opioid analgesics should be used early and ‘by the clock’, rather than ‘on-demand’, and the need for opioid analgesics should be re-evaluated every 24–48 hr, as most patients may tolerate the prescribed initial dose of the opioid analgesics, with toxic effects often emerging after 48 hr, when the parent drug and its metabolites starts accumulating. Vigilance in nursing is also required to monitor for early effects of opiate toxicity. Box 2 provides recommendations and practical points for acute pain management in CKD based on the available evidence.

Box 2. **Key points.**

- Before prescribing analgesics, consider kidney function to avoid toxicity that may occur from accumulation of the parent drug or their metabolites.
- The general principles of pain assessment and management should be adopted and analgesia should be prescribed incorporating the modified WHO analgesic ladder.
- Co-analgesics like acetaminophen should be considered “by the clock” rather than “by demand”.
- Do not write standing (or long-term) orders for opioids. Reassess the need and the dose of opioids every 24–48 hr.
- Avoid fentanyl transdermal patch in opioid-naïve patients.
