# Supplementary material for: Management of acute and post-operative pain in chronic kidney disease
Source: F1000Res. 2013 Apr 5;2:28. Originally published 2013 Jan 30. [Version 3] doi: 10.12688/f1000research.2-28.v3 (PMC3752710; doi:10.12688/f1000research.2-28.v3)
Supplement: Peer review history

# Peer review: Referee response for version 3

**Published:** 2013-04-08

**Authors:** Paul E Carns

**Affiliations:** The Department of Anesthesiology and The Division of Pain Medicine, Mayo Clinic in Rochester, Rochester, MN, USA

I have read this submission. I believe that I have an appropriate level of expertise to confirm that it is of an acceptable scientific standard.

# Peer review: Referee response for version 2

**Published:** 2013-04-03

**Authors:** Paul E Carns

**Affiliations:** The Department of Anesthesiology and The Division of Pain Medicine, Mayo Clinic in Rochester, Rochester, MN, USA

The manuscript is improved. However, the authors have not responded to my comment regarding adjuvant medications (the third bullet point in my original response to the article). The authors have still not provided a statement 'that a dose reduction may be required for patients with renal insufficiency' within the revised article. The authors only refer to drug-drug interactions and do not mention that adjuvant medications require dose adjustments with a clinical disease such as renal insufficiency. This problem is independent from any drug-drug interaction. It is important that this point is addressed by the authors in order to improve this article

I have read this submission. I believe that I have an appropriate level of expertise to confirm that it is of an acceptable scientific standard, however I have significant reservations, as outlined above.

# Peer review: Referee response for version 2

**Published:** 2013-03-18

**Authors:** Howard S Smith

**Affiliations:** Albany Medical College, New York, NY, USA

I have read this submission. I believe that I have an appropriate level of expertise to confirm that it is of an acceptable scientific standard.

# Peer review: Referee response for version 1

**Published:** 2013-02-20

**Authors:** Paul E Carns

**Affiliations:** The Department of Anesthesiology and The Division of Pain Medicine, Mayo Clinic in Rochester, Rochester, MN, USA

- The first clinical scenario includes meperidine as a drug used in the patient. The use of meperidine has significantly been reduced in the USA and is monitored as a negative finding when used in patients over 60. Thus hospitals have made efforts to reduce or eliminate its use except in treatment of rigors and given in low doses only. I am not sure if this would be the best scenario to start the article, although its issues with renal insufficiency are substantial.
- I would refrain from the use of the word 'narcotic' in your discussion in favor of the word opioid.
- In the discussion regarding 'adjuvant medications' there is no mention that nearly all these medications require dose adjustments in renal insufficiency. Since the article focuses on this topic, I believe it should be included beyond just mentioning that some patients may be using these medications.
- The last sentence in the 'Acetaminophen' section gives specific dosing recommendations to give half of the daily dose followed by round the clock dosing.  Is this opinion or should a reference be included?
- Since propoxyphene is no longer available, I would consider eliminating it from the discussion.  I am unsure if it is still being used in other countries.  If so, I guess I would leave it in.
- The Codeine/Dihydrocodeine paragraph makes no mention of the active metabolite morphine from codeine. Discussion of other drugs in this section refer to actual names of metabolites. Given that morphine was discussed earlier in the document, including this information is important in my view.

- The chart indicating % amounts in the column headed by eGFR/ml rates does not really say that the percentages indicate a percent dose reduction.  It should be made more clear even though obvious to me.  

- The methadone section says that it requires a special license to prescribe. This is not entirely true. The special license is only required when treating opioid addiction. If methadone is used for pain control, no special license is needed. I would also include that methadone is not to be used in opioid naïve patients as you do with the fentanyl patch.

I have read this submission. I believe that I have an appropriate level of expertise to confirm that it is of an acceptable scientific standard, however I have significant reservations, as outlined above.

# Peer review: Referee response for version 1

**Published:** 2013-02-19

**Authors:** Howard S Smith

**Affiliations:** Albany Medical College, New York, NY, USA

I approve this article. However, on table 3 and figure 1, oxycodone with acetaminophen is considered step 2 of  the WHO analgesic ladder but oxycodone by itself is considered step 3. I would change this.

I have read this submission. I believe that I have an appropriate level of expertise to confirm that it is of an acceptable scientific standard.
